# Gα_12_ overexpressed in hepatocellular carcinoma reduces microRNA-122 expression via HNF4α inactivation, which causes c-Met induction

**DOI:** 10.18632/oncotarget.3957

**Published:** 2015-04-29

**Authors:** Yoon Mee Yang, Chan Gyu Lee, Ja Hyun Koo, Tae Hyun Kim, Jung Min Lee, Jihyun An, Kang Mo Kim, Sang Geon Kim

**Affiliations:** ^1^ College of Pharmacy and Research Institute of Pharmaceutical Sciences, Seoul National University, Seoul, Korea; ^2^ Department of Internal Medicine, Asan Liver Center, Asan Medical Center, University of Ulsan College of Medicine, Seoul, Korea

**Keywords:** liver cancer, non-coding RNA, G protein, c-Met

## Abstract

MicroRNA-122 (miR-122) is implicated as a regulator of physiological and pathophysiological processes in the liver. Overexpression of Gα_12_ is associated with overall survival in patients with hepatocellular carcinoma (HCC). Array-based miRNA profiling was performed on Huh7 stably transfected with activated Gα_12_ to find miRNAs regulated by the Gα_12_ pathway; among them, miR-122 was most greatly repressed. miR-122 directly inhibits c-Met expression, playing a role in HCC progression. Gα_12_ destabilized HNF4α by accelerating ubiquitination, impeding constitutive expression of miR-122. miR-122 mimic transfection diminished the ability of Gα_12_ to increase c-Met and to activate ERK, STAT3, and Akt/mTOR, suppressing cell proliferation with augmented apoptosis. Consistently, miR-122 transfection prohibited tumor cell colony formation and endothelial tube formation. In a xenograft model, Gα_12_ knockdown attenuated c-Met expression by restoring HNF4α levels, and elicited tumor cell apoptosis but diminished Ki67 intensities. In human HCC samples, Gα_12_ levels correlated to c-Met and were inversely associated with miR-122. Both miR-122 and c-Met expression significantly changed in tumor node metastasis (TNM) stage II/III tumors. Moreover, changes in Gα_12_ and miR-122 levels discriminated recurrence-free and overall survival rates of HCC patients. Collectively, Gα_12_ overexpression in HCC inhibits *MIR122* transactivation by inactivating HNF4α, which causes c-Met induction, contributing to cancer aggressiveness.

## INTRODUCTION

Hepatocellular carcinoma (HCC) accounts for most of primary liver cancer cases and belongs to the leading causes of death by cancer [1]; HCC is often diagnosed at an advanced stage and has a poor prognosis due to its aggressive phenotype [2]. HCC is a malignant tumor that is frequently resistant to conventional cytostatic agent [1]. Although receptor tyrosine kinase inhibitors can be used in patients with advanced HCC, the benefits are modest with prognosis remaining poor [3]. Thus, promising therapy for advanced HCC is unavailable yet. Identification of transducers and/or cell surface receptors responsible for the acquisition of HCC malignant phenotype may be of help for the development of therapeutic strategies.

Modification of the tumor microenvironment and gain of proliferative capacities of tumor cells are the influential factors leading to poor prognosis. Heterotrimeric G proteins transmit extracellular signals from G protein-coupled receptors (GPCRs) to intracellular effector molecules. Much attention has been paid to Gα_12_ transforming *gep* oncogene because the G protein mediates growth, migration, and metastasis [4]. It is expected that Gα_12_ overexpression augments pathophysiological functions of the GPCRs interacting with sphingosine-1-phosphate (S1P), lysophosphatidic acid (LPA), thrombin, and angiotensin-II [5-7]. Moreover, levels of the ligands are often elevated in HCC and may contribute to proliferation, adhesion, invasion, and metastasis of HCC, representing poor prognosis [8]. However, little information is available on the functional role of Gα_12_ in the factors or components that leads to the aggressive phenotype of HCC.

A set of microRNAs (miRNAs) are globally dysregulated in cancer [9]. Mice with conditional deletion of Dicer-1 in hepatocytes provided the evidence that the miRNA in the liver plays a role in inflammation and cell cycle regulation [10, 11]. Furthermore, hepatocyte-specific Dicer 1 knockout mice developed spontaneous HCC [11]. In particular, miR-122 is a predominant liver-enriched miRNA, which may act as a tumor suppressor [12]. Previous studies from our laboratory reported overexpression of Gα_12_ in the patients with HCC and the association between Gα_12_ dysregulation of p53-responsive miRNAs and epithelial-mesenchymal transition (EMT) of cancer cell [13]. Because miR-122 is the most greatly and significantly suppressed by activated Gα_12_ among those down-regulated in the microarray analysis, this study investigated the effect of miR-122 dysregulation on cancer cell malignancy using cell and animal models, and human HCC samples. Here, we report c-Met as a new target of miR-122. Our findings also reveal the role of Gα_12_ pathway in the activity of hepatocyte nuclear factor 4α (HNF4α) required for the expression of *MIR122*. To verify the relationship between decrease of miR-122 by Gα_12_ and HCC progression, the levels of Gα_12_, miR-122, or c-Met were measured for human HCC samples and correlated with changes in recurrence-free and overall survival rates of the patients.

## RESULTS

### Dysregulation of miR-122 by Gα_12_

**Figure 1:**
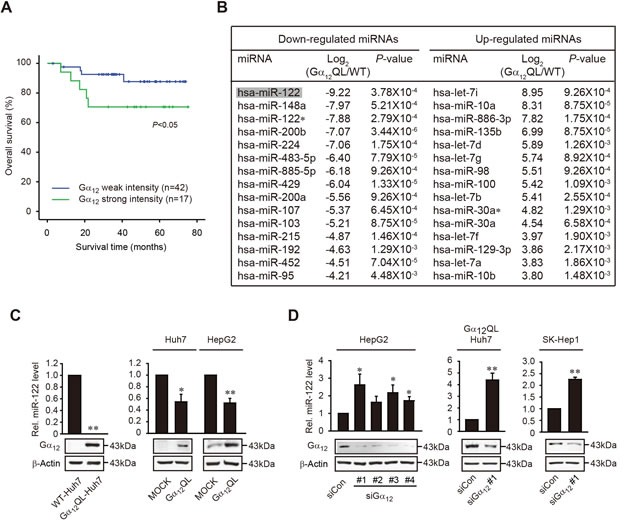
The survival rates of HCC patients in association with Gα_12_ intensity and miR-122 repression by activated mutant of Gα_12_ **A.** Kaplan-Meier analysis of overall survival in patients with HCC according to weak or strong Gα_12_ expression. Fifty-nine HCC patient specimens were subjected to immunoblottings for Gα_12_, as shown in the previous study [13]. The weak and strong intensities were defined by Gα_12_ expression in HCC/NT_avg_ ≤3-fold; and Gα_12_ expression in HCC/NT_avg_ >3-fold, respectively. *P* value was generated by a Breslow test. **B.** Top 15 microRNAs most significantly down-regulated or up-regulated in Gα_12_ QL-Huh7 cells compared to WT-Huh7. Microarray analyses were done to assess alterations in miRNAs expression in Gα_12_QL-Huh7. Log_2_ (Gα_12_QL/WT) ratio of differentially expressed top 15 miRNAs that reached statistical significance by *t*-test (*P*<0.01) and further confirmed by SAM test (q<5%). **C.** The effect of Gα_12_QL on miR-122 level. qRT-PCR assays were performed on the cells stably (left) or transiently transfected with Gα_12_QL (right). U6 small nuclear RNA was used as a normalizing reference for miRNA. Gα_12_QL overexpression was verified by immunoblottings. **D.** The effect of Gα_12_ knockdown on miR-122 levels. HepG2 cells were transfected with control siRNA (siCon) or four different siRNAs directed against Gα_12_ (siGα_12_), whereas Gα_12_QL-Huh7 or SK-Hep1 cells were done with siGα_12_ #1. For **C.**-**D.** data represent the mean±S.E. (*n* = 3, *P < 0.05, **P < 0.01, significant compared with the respective control).

To evaluate whether Gα_12_ expression is associated with prognosis of HCC, we first carried out a survival analysis using electronic medical records and 59 primary human HCC samples stratified based on Gα_12_ levels measured as in the previous study [13]. The cutoff for strong Gα_12_ intensity was set at ‘>3-fold’ difference in HCC/NT_avg_. The intensity of Gα_12_ was strongly detected in 28.8% (17/59) of the cancerous samples and significantly correlated with shorter overall survival in HCC patients (Figure 1A). Next, we compared miRNA microarray profiles using wild-type (WT) Huh7 and Huh7 cells stably transfected with a constitutively active mutant of Gα_12_ (Gα_12_QL-Huh7) (Figure 1B). In our finding, activated Gα_12_ most substantially and significantly repressed miR-122: either stable or transient transfection with Gα_12_QL reduced the levels of mature form of miR-122 in Huh7 or HepG2 cells (Figure 1C). To verify the effect of siRNA knockdown of Gα_12_ on miR-122 expression, we examined the effects of four different siRNAs on the basal Gα_12_ expression in HepG2 cells (Figure 1D). Transfection with each of the four different siRNAs caused sufficient knockdown of Gα_12_. Consistently, miR-122 levels were significantly decreased in the samples except siRNA #2 (siGα_12_ #2). Based on the targeting efficacy on miR-122 expression, siGα_12_#1 was selected in the subsequent experiments. In Gα_12_QL-Huh7 or SK-Hep1, siRNA knockdown of Gα_12_ promoted increase of miR-122. Our data indicates that Gα_12_ overexpressed in liver cancer mostly greatly dysregulates the expression of miR-122.

### Inhibition of c-Met by miR-122

**Figure 2:**
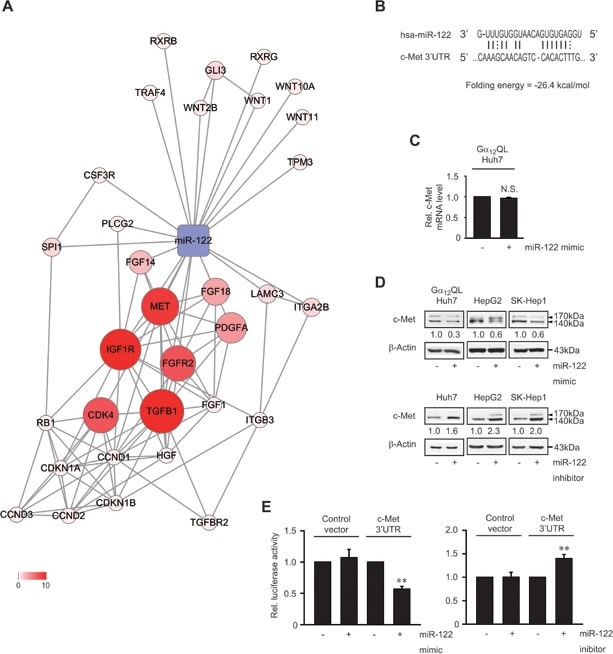
c-Met inhibition by miR-122 **A.** An integrative network of putative or validated targets of miR-122. Nodes represent genes/proteins, whereas edges represent interactions. Colors and node size reflect the number of interactions. **B.** Prediction of miR-122 binding to the 3′UTR of human c-Met mRNA. **C.** The effect of miR-122 mimic treatment on c-Met transcript level. N.S., not significant. **D.** The effect of miR-122 modulations on c-Met expression. Immunoblottings for c-Met were done on the lysates of Gα_12_QL- or WT-Huh7, HepG2, or SK-Hep1 cells transfected with miR-122 mimic, miR-122 inhibitor or the respective negative control. **E.** The effect of miR-122 modulations on pEZX-c-Met-3′UTR luciferase activity. Luciferase activities were measured on HEK293 cells transfected with miR-122 mimic, miR-122 inhibitor or the respective negative control in combination with pEZX-control or pEZX-c-Met-3′UTR. Data represent the mean±S.E. (*n* = 3, significantly different from the respective control, **P < 0.01).

Having identified the most evident decrease of miR-122 by the activated form of Gα_12_, we searched for the target of miR-122 as a protein possibly implicated in the aggressiveness of HCC. Bioinformatic analyses using Microcosm program enabled us to select the targets putatively regulated by miR-122. Among the putative, but yet unidentified, targets of miR-122, c-Met was the most enriched interacting molecule of the pathway in cancer (Figure 2A). We found a putative miR-122 binding site within the 3′-untranslated region (3′UTR) of c-Met mRNA using RNA 22 program (Figure 2B). To clarify the role of miR-122 in regulating c-Met, *in vitro* functional assays were done after enhancing or silencing the miRNA. Transfection with miR-122 mimic unchanged c-Met mRNA level (Figure 2C). miR-122 mimic transfection notable decreased c-Met protein levels in three different cell lines, whereas miR-122 inhibitor increased them (Figure 2D). Consistently, miR-122 mimic diminished luciferase expression from pEZX-c-Met-3′UTR luciferase construct comprising the c-Met 3′UTR region (Figure 2E). Transfection with miR-122 inhibitor enhanced the 3′UTR reporter activity. These results show that miR-122 directly inhibits c-Met translation by targeting the 3′UTR region.

### c-Met overexpression by activated Gα_12_ and the effects of LPA and S1P on miR-122 and c-Met expression

**Figure 3:**
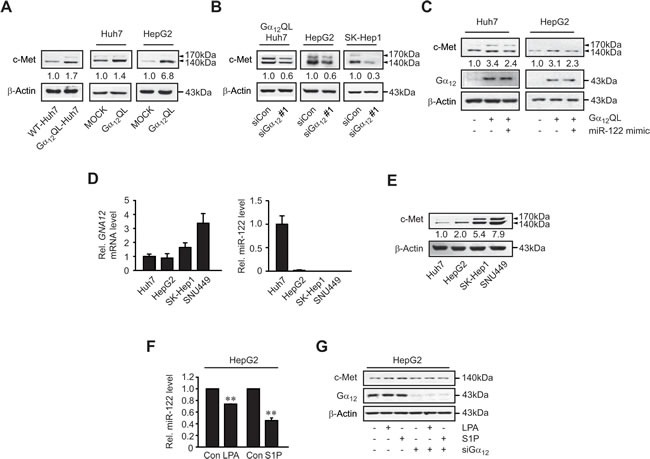
c-Met induction by Gα_12_ and the effects of LPA and S1P on miR-122 expression **A.** The effect of Gα_12_QL transfection on c-Met levels. **B.** The effect of siRNA knockdown of Gα_12_ on c-Met levels. **C.** The effect of miR-122 mimic on c-Met induction by Gα_12_QL. WT- or Gα_12_QL-Huh7 cells were transfected with control mimic or miR-122 mimic. HepG2 cells were co-transfected with either pCDNA3 or Gα_12_QL and either control mimic or miR-122 mimic. **D.** qRT-PCR assays for Gα_12_ or miR-122 in liver tumor cell lines. Data represent the mean±S.E. (*n* = 3). **E.** Immunoblottings for c-Met. **F.** qRT-PCR assays for miR-122. MiR-122 levels were measured in HepG2 cells treated with 20 μM lysophosphatidic acid (LPA) or 1 μM sphingosine 1-phosphate (S1P) for 12 h. Data represent the mean±S.E. (*N* = 3, significantly different from vehicle-treated control, **P < 0.01). **G.** Immunoblotting for c-Met. HepG2 cells were transfected with control siRNA or Gα_12_ siRNA for 48 h and were continuously treated with either LPA or S1P as described in panel **F.**

Having identified the link between miR-122 and c-Met downstream of Gα_12_, we next confirmed the effect of Gα_12_ modulations on c-Met. Either stable or transient transfection of Huh7 (or HepG2) cells with Gα_12_QL increased c-Met levels (Figure 3A), whereas knockdown of Gα_12_ reduced them (Figure 3B). In addition, transfection with miR-122 mimic diminished the induction of c-Met by Gα_12_QL (Figure 3C).

Previously, we observed that Gα_12_ is expressed to greater levels in mesenchymal cell lines (SK-Hep1 and SNU449) than epithelial cell lines (Huh7 and HepG2) [13]. To further link Gα_12_ and miR-122 physiologically, we examined their levels in a panel of human HCC cell lines. The *GNA12* transcript levels were both higher in the latter than the former (Figure 3D, left). Consistently, miR-122 contents were lower in the mesenchymal cell lines (Figure 3D, right). The c-Met levels were also higher in SK-Hep1 and SNU449 than Huh7 and HepG2, as were *GNA12* mRNA levels (Figure 3E). All of these results indicate that increased levels of Gα_12_ causes the induction of c-Met by deregulating miR-122.

Many GPCRs facilitate tumor progression [14]. The GPCRs including Edg receptors (LPA and S1P receptors) activate the Gα_12_ signaling pathway. Increase of LPA contributes to tumorigenesis and tumor progression [15]. Ligand activation of S1P_1_, S1P_3_, and S1P_5_ also causes NF-κB-mediated COX-2 induction via Gα_12_, potentially promoting tumor growth [5]. We found that treatment of HepG2 cells with LPA (12 h) moderately reduced miR-122 levels (Figure 3F). S1P treatment also significantly decreased miR-122 level (Figure 3F). siRNA knockdown of Gα_12_ diminished the ability of LPA or S1P to induce c-Met (Figure 3G), supporting the concept that Gα_12_ transduces GPCR signals for decrease of miR-122 presumably in tumor microenvironments.

### Gα_12_ inactivation of HNF4α necessary for the basal expression of MIR122

**Figure 4:**
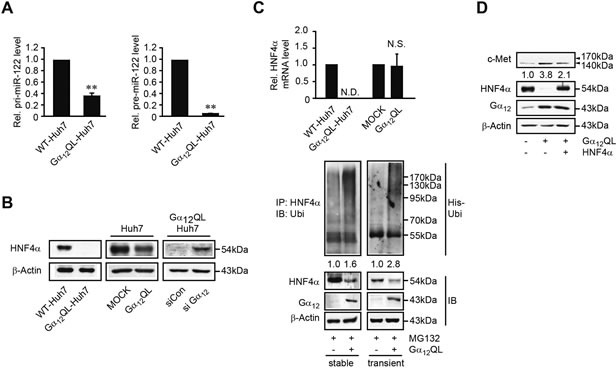
Repression of HNF4α-mediated miR-122 transcription by Gα_12_ **A.** qRT-PCR assays for primary or precursor form of miR-122. Data represent the mean±S.E. (*n* = 3, **P < 0.01, significantly different from WT-Huh7). **B.** The effect of Gα_12_ modulations on HNF4α level. HNF4α was immunoblotted on the lysates of Huh7 cells stably (left) or transiently transfected with Gα_12_QL (middle) or Gα_12_QL-Huh7 cells transiently transfected with siGα_12_ (right). **C.** qRT-PCR assays for HNF4α mRNA (upper). Increase in HNF4α ubiquitination by Gα_12_(lower). Huh7 cells transfected with the plasmid encoding His-Ubi were treated with 10 μM MG132 for 3 h. HNF4α immunoprecipitates were immunoblotted with anti-ubiquitin antibody. **D.** The effect of HNF4α overexpression on c-Met induction by Gα_12_QL. WT- or Gα_12_QL-Huh7 cells were transfected with Mock or a construct encoding for HNF4α for 24 h.

To precisely define the underlying basis of miR-122 repression by Gα_12_ signaling, the levels of miR-122 primary transcript and of its precursor form were measured in Gα_12_QL-Huh7 cells. Activated Gα_12_ decreased both the primary and the precursor forms of miR-122 transcript levels (Figure 4A, left and right), suggesting that activated Gα_12_ inhibits *MIR122* gene transcription. HNF4α, a transcription factor belonging to the HNF family members, may regulate the *MIR122* gene [16]. As a continuing effort to find the basis of miR-122 dysregulation by Gα_12_, we assessed the enhancing or silencing effect of Gα_12_ on HNF4α; transfection with Gα_12_QL diminished HNF4α level, whereas siRNA knockdown of Gα_12_ accumulated it (Figure 4B). In quantitative real-time polymerase chain reaction (qRT-PCR) analysis, stable transfection of Huh7 cells with Gα_12_QL nullified HNF4α mRNA levels (Figure 4C, upper). However, transient transfection of Gα_12_QL did not change them, suggesting that the decrease in HNF4α mRNA in Gα_12_QL-Huh7 cells may have resulted from the adaptive change. Next, we assessed whether Gα_12_ facilitates post-translational modification of HNF4α for destabilization. In Huh7 cells, activated Gα_12_ increased HNF4α ubiquitination for degradation (Figure 4C, lower). In addition, Gα_12_QL overexpression promoted c-Met level, which was reversed by overexpression of HNF4α (Figure 4D). These results provide evidence that Gα_12_ decreases miR-122 levels by inhibiting HNF4α activity, which may contribute to c-Met up-regulation.

### Inhibition of cancer cell aggressiveness by miR-122

**Figure 5:**
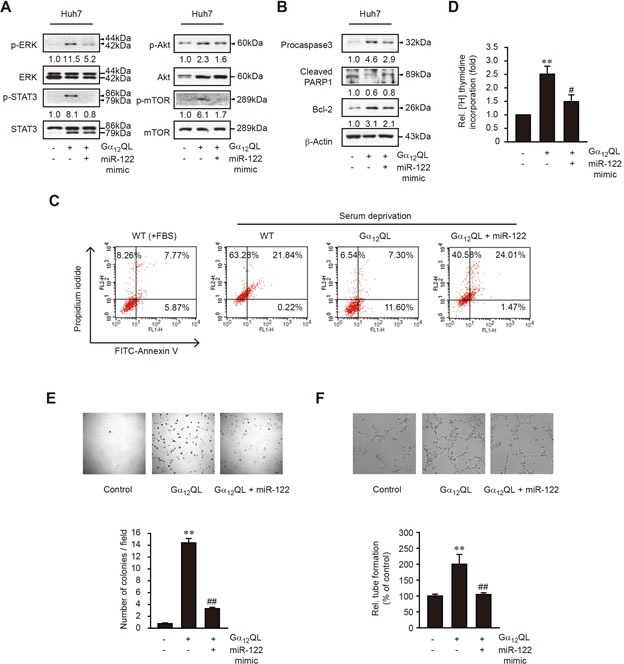
The effect of miR-122 overexpression on tumor cell survival, proliferation, colony formation, and tube formation in Gα_12_QL-Huh7 cells **A.** The effect of miR-122 mimic transfection on signaling pathways downstream from c-Met. WT- or Gα_12_QL-Huh7 cells were transfected with control mimic or miR-122 mimic for 48 h. **B.** The effect of miR-122 mimic on the proteins related with apoptosis or survival. **C.** The effect of miR-122 mimic transfection on the cell apoptosis caused by serum deprivation. The cells were transfected as in panel **A.** for 48 h, and were incubated in DMEM containing 10% FBS or serum-free DMEM for additional 24 h. Early or late apoptosis was assessed by flow cytometry after staining with FITC-Annexin V/PI. The cells in the lower right quadrant indicate FITC-Annexin V^+^/PI^−^ (early apoptosis), whereas those in the upper right quadrant do Annexin V^+^/PI^+^ (late apoptosis). Results are representative of three independent experiments. **D.** [Methyl-^3^H]-thymidine incorporation assay. DNA synthesis rate was measured in Huh7 cells transfected with control, or miR-122 mimic for 48 h, and subsequently exposed to [methyl-^3^H]-thymidine for 8 h. **E.** Colony formation assay. Data are representative of three independent experiments, with 5 fields counted per experiment. **F.** Endothelial cell tube formation assay. Micrographs of tube formation by BAECs grown on Matrigel in a conditioned medium collected from Huh7 cells transfected as in panel **A.** upper. Branches formed during tubulogenesis were counted (lower). For **D.**-**F**, data represent the mean±S.E. (*n* = 3, significantly different from WT-Huh7 transfected with control mimic, **P < 0.01; significantly different from Gα_12_QL-Huh7 transfected with control mimic, ^#^*P* < 0.05, ^##^*P* < 0.01).

c-Met activation triggers a variety of cellular responses, including survival, proliferation, and angiogenesis [17]. Multiple signaling pathways such as the mitogen-activated protein kinase/extracellular signal-regulated kinases (ERK), signal transducer and activator of transcription 3 (STAT3), and Akt/mammalian target of rapamycin (mTOR) pathways are involved in tumor biology [18]. We investigated the effect of activated Gα_12_ and miR-122 mimic transfection on the signaling pathways of c-Met using a cell model. Gα_12_QL transfection promoted phosphorylation of ERK, STAT3, Akt, and mTOR in Huh7 cells, whereas transfection with miR-122 mimic abrogated this effect (Figure 5A). Evasion of apoptosis is a crucial event during malignant transformation [19]. To further understand the mechanism of Gα_12_ oncogenic activity, we assessed the role of Gα_12_-miR-122 pathway in tumor cell death. Transfection of the cells with miR-122 mimic diminished the ability of Gα_12_QL to increase procaspase 3 and B-cell lymphoma 2 (Bcl-2) levels, but enhanced poly[ADP-ribose]polymerase 1 (PARP1) cleavage (Figure 5B), supporting the induction of cell death. Adaptation to nutrient deprivation is presumed to be one of the prerequisites for cancer cells to survive in the tumor microenvironment [20]. At 3 days after serum starvation, up to 21% of WT-Huh7 cells underwent late apoptosis while stable transfection of Gα_12_QL significantly decreased the population of Annexin V^+^/PI^+^, indicative of rescue of the cells from the loss of membrane integrity and death (Figure 5C). As compared with control miRNA mimic, miR-122 mimic transfection significantly (∼3-fold) facilitated the apoptosis of Gα_12_QL-Huh7 cells caused by serum starvation for 3 days, strengthening the concept that miR-122 plays a critical role in sensitizing cancer cell apoptosis to stimulus.

In Gα_12_QL-Huh7 cells, DNA synthesis rate was much augmented as compared to control, which was repressed by transfection with miR-122 mimic (Figure 5D). Consistently, stable transfection with Gα_12_QL promoted Huh7 cell proliferation under anchorage-independent condition, and this effect was attenuated by miR-122 mimic transfection (Figure 5E). To explore the biological significance of Gα_12_ signaling in angiogenesis, we evaluated capillary tube formation of bovine aortic endothelial cells (BAECs) using the conditioned media collected from WT-Huh7 or Gα_12_QL-Huh7 cells transfected with control mimic or miR-122 mimic. A clear difference was found in capillary tube formation after miR-122 mimic transfection (Figure 5F).

### Gα_12_ knockdown effects in a tumor-xenograft model

**Figure 6:**
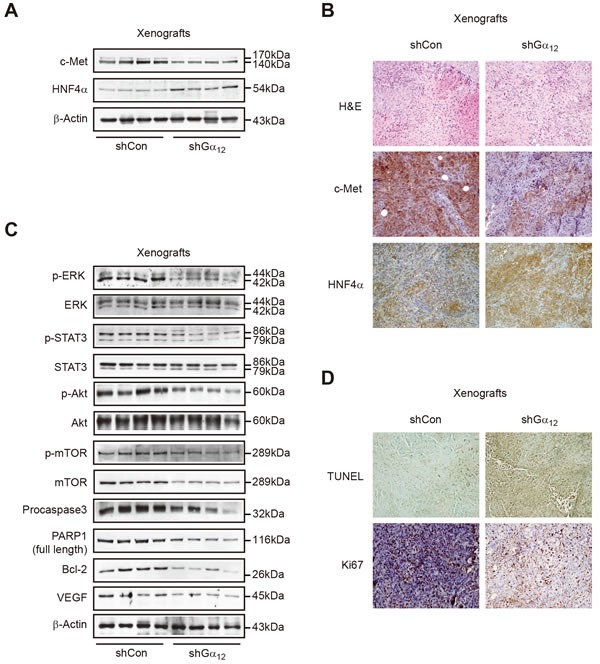
The effect of sustained depletion of Gα_12_ using shRNA on tumor cell survival and protein markers in mice **A.** Immunoblottings for c-Met and HNF4α in subcutaneous Gα_12_-depleted SK-Hep1 tumor. Data shown are representative of shCon-SK-Hep1 (*n* = 9) or shGα_12_-SK-Hep1 (*n* = 8) groups. **B.** Immunohistochemistry for c-Met and HNF4α in the xenograft tumor tissues (×200, representative figures were shown; *n* = 4 in each group). **C.** Representative immunoblottings for cell-signaling molecules. **D.** Representative TUNEL and Ki67 staining of the xenograft tumor tissues.

In a previous study, shRNA inhibition of Gα_12_ resulted in a profound anti-tumor effect in a tumor xenograft animal model using SK-Hep1, a mesenchymal type of tumor cell [13]. These samples were used in the present study to further evaluate Gα_12_ impact on tumor aggressiveness. Immunoblottings and immunohistochemistry showed that shRNA knockdown of Gα_12_ suppressed c-Met expression, but increased HNF4α levels in the xenograft tumor tissue (Figure 6A and B). Consistently, the phosphorylation of ERK, STAT3, Akt and mTOR, which are downstream molecules from c-Met, was all diminished in the tumors depleted of Gα_12_ (Figure 6C). Similarly, procaspase 3, PARP1, Bcl-2, and vascular endothelial growth factor (VEGF) levels were attenuated (Figure 6C). Moreover, knockdown of Gα_12_ facilitated tumor cell death, as indicated by an increase in terminal transferase-mediated dUTP nick-end labeling (TUNEL) staining intensity (Figure 6D). Similarly, Ki67 staining intensities were also reduced in the xenograft tumor samples (Figure 6D). Our results provide strong evidence that Gα_12_ inhibition impedes the survival and growth advantage of mesenchymal liver tumor cells, which may be associated with c-Met suppression.

### Association of Gα_12_/miR-122/c-Met changes with HCC patient survival

**Figure 7:**
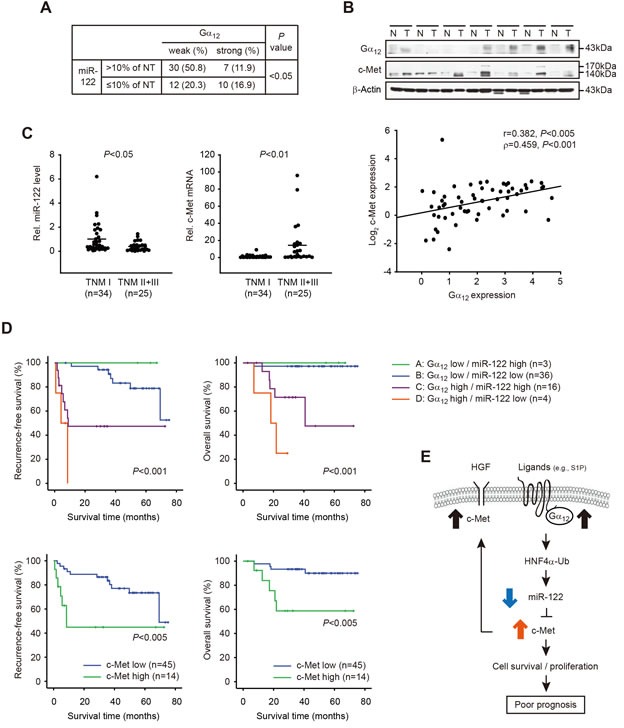
Clinical outcomes of HCC patients in association with weak or strong Gα_12_ expression, miR-122 dysregulation, and c-Met induction **A.** Association between Gα_12_ and miR-122 repression in patients with HCC. The samples were divided into two groups according to the results of qRT-PCR assays for miR-122; miR-122 down-regulation (≤10%) was found in 37% of HCC samples (22 of 45). Gα_12_ intensities were assessed as in Figure 1A. The weak and strong intensities were defined by Gα_12_ expression level in HCC/NT_avg_ ≤3-fold; and Gα_12_ expression level in HCC/NT_avg_ >3-fold, respectively. The data were analyzed by Chi-square test. **B.** Correlation between Gα_12_ and c-Met levels in HCC. Immunoblottings for Gα_12_ and c-Met were carried out on the homogenates of 59 pairs of HCC samples and were normalized to those of β-actin. Shown above are the representative blots for HCC (T) and non-tumorous (N) samples. Two variables were correlated by Pearson (r) or Spearman (ρ) correlation coefficients (lower). **C.** The relative levels of miR-122 or c-Met in the patients with HCC in different TNM stages. The line indicates the mean value. Statistical analysis was done using Student's *t*-test. **D.** Kaplan-Meier survival curves for 9 HCC patients with respect to Gα_12_ and miR-122 expression (upper). Tumor samples were divided into four groups according to mean fold changes (T/N) of Gα_12_ or miR-122. Recurrence free survival or overall survival of patients with low (≤2-fold) or high (>2-fold) c-Met transcript levels (lower). *P* value was generated by log-rank test and Breslow test. **E.** A schematic diagram illustrating the proposed mechanism by which Gα_12_ dysregulation of miR-122 contributes to poor prognosis in patients with HCC.

To further explore the relationship between Gα_12_ and miR-122 (or c-Met), we examined the expression of miR-122 and c-Met in tissues from 59 human primary HCC and matched non-tumorous (NT) tissues. The chi-square test showed a significant association between Gα_12_ and miR-122 expression (Figure 7A), whereas the Pearson or Spearman analysis failed to do so (Supplementary Figure 1). Immunoblottings confirmed overexpression of Gα_12_ and c-Met in the HCC compared to adjacent NT (Figure 7B, upper). In addition, a positive and significant correlation existed between Gα_12_ and c-Met in the samples (Figure 7B, lower). In a subgroup analysis, we found that miR-122 repression and c-Met induction were distinct in TNM stage II and III tumors (n=25) compared to TNM stage I tumors (n=34) (Figure 7C), consolidating the clinical relevance of miR-122 and c-Met changes with tumor stage progression (i.e., aggressive feature). Moreover, HCC patients with high Gα_12_ and low miR-122 had the poorest prognosis (i.e., the lowest overall survival and highest probability of tumor recurrence), whereas those with low Gα_12_ and high miR-122 had the best outcomes (Figure 7D, upper). Also, we verified that the patients with high c-Met in HCC had shorter overall survival and higher possibilities of tumor recurrence as compared with the patients with low c-Met in HCC (Figure 7D, lower). These results support the conclusion that Gα_12_ overexpression causes miR-122 dysregulation, promoting c-Met induction, which may deteriorate the prognosis, recurrence-free and overall survival rates of HCC patients.

## DISCUSSION

miR-122 levels in the liver amount to 135,000 copies per normal human hepatocyte [21], representing 72% of all miRNAs. miR-122 is necessary for the control of lipid and glucose metabolism, and other physiological activities in the liver [22, 23]. *Mir122a*−/− mice spontaneously develop liver tumors [24]. In addition, miR-122 is frequently under-expressed in human HCC [25]. Moreover, the loss of miR-122 alters hepatic phenotype, assisting gain of metastatic properties, which strengthens the concept that miR-122 may be an intrinsic tumor suppressor gene in the liver [24-26]. Nevertheless, the upstream regulator of miR-122 and the basis underlying miR-122 dysregulation in HCC had been elusive. Our results shown here demonstrate for the first time that Gα_12_ overexpressed in the tumor decreases miR-122, accounting for cancer aggressiveness and poor prognosis of the patients with HCC.

Gα_12_ may also play a role in the progression of cancer malignancy through other pathways. EMT has been implicated in tumor invasion and metastasis [27]. Previously, we found that Gα_12_ was overexpressed in HCC, which caused ZEB1 induction through p53-responsive miRNAs deregulation, promoting EMT of liver tumor [13]. It is noteworthy that hepatocyte growth factor (HGF) elicits mitogenic, and morphogenic properties [28, 29], and reduces the expression of E-cadherin with increase of N-cadherin [30]. Promotion of cancer EMT program and cell migration by HGF depends on c-Met [31]. A novel finding of this study is the identification of c-Met as a new target of miR-122, as evidenced by the outcomes of *in vitro* functional assays using a construct comprising c-Met 3′UTR and bioinformatic analysis. Several miRNAs including miR-103 and -203 also affect c-Met expression [32]. Of the miRNAs normally enriched in hepatocytes, activated Gα_12_ most greatly and significantly decreased miR-122 levels. Since miR-122 is the most abundant in the liver, the repression of miR-122 by Gα_12_ would greatly alter cell biology in association with c-Met-mediated aggressiveness (e.g., anti-apoptosis, proliferation, and angiogenesis). Because miRNAs have overlapping targets, other targets of miR-122 including cyclin G1 and ADAM17 may additionally be involved in HCC pathogenesis [25, 33]. Hence, c-Met and others would work together for tumor malignancy [33].

miRNAs are transcribed by RNA polymerase II as pri-miRNAs which undergo nuclear export and cytoplasmic cleavage to generate mature forms [34]. Pri-miR-122 had been identified as a non-coding RNA, *hcr* [21]. The expression of miR-122 relies on liver-enriched transcription factors in the developing liver or cell lines [16]. In particular, HNF4α is abundantly expressed in the liver, and directly binds to the promoter region of the *MIR122* gene [35]. In the present study, activated Gα_12_ decreased the levels of primary and precursor forms of miR-122, supporting the role of Gα_12_ in inhibiting *MIR122* transcription. An important finding of our study is the ability of Gα_12_ to negatively control HNF4α, a transcription factor required for the constitutive *MIR122* gene expression [35]. Gα_12_ transduces c-Jun N-terminal kinases (JNK)-dependent signaling for Nrf2 and IκBα ubiquitination [5, 37]. It also decreased p53 and FOXO1 levels through the induction of MDM2, an E3 ubiquitin ligase [13, 38]. Since Gα_12_ activates JNK1 [36], miR-122 repression by Gα_12_ may be associated with JNK1-dependent inhibition of HNF4α. Our data showing an increase in HNF4α ubiquitination by Gα_12_ supports the idea that JNK1 activated by Gα_12_ may decrease miR-122 through the inhibitory phosphorylation and ubiquitination of HNF4α [23].

There has been an increasing interest which focuses on heterogeneous inter-receptor networks. A majority of GPCRs has growth-promoting activity by trans-activating receptor tyrosine kinases [40]. Our result shown here identifies c-Met as a novel target of the Gα_12_ pathway. Ligand activation of c-Met causes phosphorylation of two tyrosine residues, which activates Ras/MAPK and PI3K/AKT pathways through recruitment of adaptor proteins, promoting tumor growth and metastasis [17]. Since certain GPCR ligands transactivate c-Met [41], this may act as a point of convergence for different cell-surface receptors. Gα_12_ increases the activities of Rho/Rac-dependent AP-1 and others (e.g., STAT3) [42, 43], conferring on cells the ability to recruit multiple receptors, non-receptors, and Ser/Thr kinases for neoplastic transformation and progression. Hence, Gα_12_ overexpression in HCC makes a positive feed-forward loop in activating signaling such as ERK1/2, STAT3, Akt, and mTOR through up-regulation of c-Met as a consequence of decrease of miR-122 in the tumor tissue.

The balance between proliferation and apoptosis is frequently disrupted in tumor tissues, and the acquisition of abnormal growth rates and anchorage-independent growth advances tumor malignancy [44]. In our findings, Gα_12_QL transfection prevented apoptosis, but promoted soft agar colony growth of tumor cells. The results that Gα_12_QL transfection initiated capillary tube formation (i.e., a late stage of angiogenesis) and this effect was antagonized by miR-122 mimic transfection support the notion that miR-122 acts as a suppressor of liver tumor progression in severity. This concept is re-enforced by the finding that Gα_12_ knockdown not only attenuated c-Met, the downstream signals, and Ki67 intensities in a xenograft model, but increased tumor cell death.

In patients with HCC, Gα_12_ levels correlated with either decrease of miR-122 or c-Met induction in the HCC samples. Moreover, these changes correlated with TNM stages, suggestive of the role of miR-122 and c-Met dysregulation in the tumor stage progression. Similarly, miR-122 was depressed in a subset of HCC harboring c-Met signature [26]. Our findings support the reciprocal link between miR-122 and c-Met expression downstream from increase of Gα_12_, extending basic scientific information to clinical arena. The result also supports the role of Gα_12_ as an independent prognostic factor for tumor recurrence particularly in combination with low miR-122. Overall, our findings provide an insight into (1) the inhibitory role of Gα_12_ in miR-122 targeting c-Met, and (2) the crosstalk between GPCR and c-Met in HCC, implying that intervention of the Gα_12_ pathway may be of help to improve c-Met-targeted therapy.

## MATERIALS AND METHODS

### Human liver samples

A total of 59 paired samples of HCC and NT tissue were obtained from the Bio-Resource Center at the Asan Medical Center, Seoul, Korea [13]. Informed consent was provided in accordance with the ethical guidelines of the 1975 Declaration of Helsinki. Written informed consent was obtained from all patients. The study protocol was approved by institutional review board of Asan Medical Center (#2012-0133)

### Microarray

The purified labeled miRNA probes were hybridized to 8×15 K human miRNA microarrays from Agilent Technologies previously [13]. Our dataset is available from NCBI's Gene Expression Omnibus (accession number GSE44079).

### Materials

Antibodies recognizing Gα_12_, HNF4α, caspase-3, PARP1, Bcl-2, and VEGF were obtained from Santa Cruz Biotechnology (Santa Cruz, CA). β-Actin and ubiquitin antibodies, propidium iodide (PI), and other reagents were supplied from Sigma-Aldrich (St. Louis, MO). Antibodies directed against c-Met, ERK, phospho-ERK (Thr202/Tyr204), STAT3, phospho-STAT3 (Tyr705), mTOR, phospho-mTOR (Ser2448), Akt, and phospho-Akt (Ser473) were purchased from Cell Signaling Technology (Danvers, MA). The anti-Ki67 antibody was obtained from Diagnostics Biosystems (Pleasanton, CA). Horseradish peroxidase-conjugated goat anti-rabbit and goat anti-mouse IgGs were from Zymed Laboratories (San Francisco, CA). MG132 was provided from Calbiochem (La Jolla, CA). Fluorescein isothiocyanate (FITC)-Annexin V and BD Matrigel^TM^ basement membrane matrix growth factor reduced phenol red-free were supplied from BD Biosciences (San Jose, CA). [Methyl-^3^H]-thymidine was obtained from Amersham Biosciences (Buckinghamshire, UK). DeadEnd^TM^ Colorimetric TUNEL System was purchased from Promega (Madison, WI).

### Cell culture

Human HCC cell lines, Huh7, SK-Hep1, and SNU449 were provided from Korean Cell Line Bank (KCLB, Seoul, Korea). HepG2 cell line was supplied from American Type Culture Collection (Manassas, VA). Huh7 cells stably expressing an active mutant of Gα_12_ (Gα_12_Q229L, Gα_12_QL) and SK-Hep1 cells stably expressing shRNA against Gα_12_ were established, as described previously [13, 39]. The cells were maintained in a growth medium containing Dulbecco's Modified Eagle's Medium (DMEM), 10% fetal bovine serum (FBS), and 5% penicillin-streptomycin at 37°C in a humidified atmosphere containing 5% CO_2_. BAECs were a generous gift of Drs. HJ Kim and KC Chang (Gyeongsang National University, Jinju, Korea). BAECs were cultured in EGM-MV media (Lonza, Walkersville, MD).

### Transient transfection

Active mutant of Gα_12_ was kindly provided from Dr. N. Dhanasekaran (The University of Oklahoma Health Sciences Center). Scrambled control siRNA, or siRNAs specifically directed against Gα_12_ (siGα_12_ #1) were purchased from Santa Cruz Biotechnology (Santa Cruz, CA). All duplexes were synthesized as 21-mers with 3′dTdT overhangs. Sequences of synthetic siRNAs were as follows: siGα_12_ #2, 5′CAACATCCTCAAGGGCTCAdTdT3′; siGα_12_ #3, 5′CCAAGGGAATTGTGGAGCAdTdT3′; and siGα_12_ #4, 5′CCAGCGAGCTCTAGGCAAAdTdT3′. Sense sequence is only shown. miR-122 mimic and its negative control were synthesized by GenePharma (Shanghai, China). The miR-122 inhibitor and negative control were purchased from Dharmacon (Lafayette, CO). The transfection with the plasmid of interest (1 μg), siRNA (100 nM), miRNA mimic (100 nM) or inhibitor (100 nM) were done using FuGENE^®^ HD Reagent in accordance with manufacturer's procedure (Roche, Indianapolis, IN).

### Real-time PCR assays

Total RNA was extracted using Trizol (Invitrogen, Carlsbad, CA). qRT-PCR assays for miRNAs were performed using miScript SYBR Green PCR kit (Qiagen, Valencia, CA), whereas those for mRNAs were done using LightCycler^®^ DNA master SYBR Green-I kit (Roche, Mannheim, Germany) according to the manufacturer's instruction.

### Immunoblot analysis

Sodium dodecyl sulfate-polyacrylamide gel electrophoresis was carried out using whole cell lysates or liver homogenates according to the previous method [23]. The proteins of interest were visualized using an ECL chemiluminescence detection kit (Amersham Biosciences, Amersham, UK). At least three independent experiments were performed. Scanning densitometry of the immunoblots was performed with the Image Scan and Analysis System (Alpha Innotech Corp, San Leandro, CA). The band intensity was measured using Adobe Photoshop (Adobe Systems, Inc., San Jose, CA).

### Bioinformatic analyses

The potential targets of miR-122 were extracted from Microcosm program (http://www.ebi.ac.uk/enright-srv/microcosm/htdocs/targets/v5/). Predicted (or validated) targets of miR-122 were subjected to KEGG enriched pathway analysis (pathway in cancer) using the DAVID 6.7 (http://david.avcc.ncifcrf.gov/ref) online bioinformatics tool. Gene interaction analysis between the clustered genes was achieved according to STRING v9.1 database (http://string-db.org). Further visualization was done using Cytoscape 3.0.0 software.

### 3′UTR luciferase assay

The miRNA 3′UTR target clone (Luc-MET-3′UTR) was purchased from GeneCopoeia (Rockville, MD), which contains renilla luciferase as internal control fused downstream to a firefly luciferase. The cells were co-transfected with control or c-Met 3′UTR luciferase vector and miR-122 mimic (or inhibitor) or its relative control using FuGENE^®^ HD Reagent (Roche, Indianapolis, IN). After 48 h of transfection, firefly and renilla luciferase activities were measured using Luc-Pair miR Luciferase Assay (GeneCopoeia) according to the manufacturer's protocols.

### Immunoprecipitation assay

To assess HNF4α ubiquitination, cells were transfected with a plasmid encoding His-tagged ubiquitin (His-Ubi) for 6 h. Transfected cells were then maintained in Eagle's minimum essential medium containing 1% FBS for 18 h. Cell lysates were incubated with anti-HNF4α antibody overnight at 4°C. After immunoprecipitation, the antigen-antibody complex was precipitated following incubation for 2 h at 4°C with protein G-agarose. The immune complex was solubilized in 2×Laemmli buffer and boiled for 5 min. The samples were immunoblotted with anti-ubiquitin antibody.

### Flow cytometric analysis of apoptosis

Apoptosis was analyzed by the FITC-Annexin V plus PI staining method. The transfected cells were harvested by trypsinization. After washing with phosphate buffered saline (PBS) containing 1% FBS, the cells were stained with 5 μl FITC-Annexin V and 2 μg/ml PI. The fluorescence intensity in the cells was assessed using BD FACSCalibur II flow cytometer and the CellQuest software (BD Biosciences, San Jose, CA). In each analysis, 20,000 gated events were recorded.

### Thymidine incorporation

The rate of DNA synthesis was measured using [methyl-^3^H]-thymidine incorporation assay. Post-confluent cells in 12-well plates were incubated with 10% FBS for 24 h after transfection. The cells were pulse-labeled with 1 μCi/ml [methyl-^3^H]-thymidine for 8 h, washed with PBS twice, fixed with 5% trichloroacetic acid for 30 min, and finally dissolved in 0.5 N NaOH containing 0.1% sodium dodecyl sulfate. The radioactivity was measured using a liquid scintillation counter (PerkinElmer, Waltham, MA).

### Agarose colony-forming assay

A total of 5×10^3^ cells were suspended in 1.5 ml of DMEM medium containing 10% FBS and 0.3% agarose, were plated in 35-mm 0.6% base agar dishes, and were incubated in 37°C and 5% CO_2_ incubator for 3 weeks. Then, the cells were fixed in 3.7% paraformaldehyde and stained with 0.005% crystal violet. Colonies (diameter of more than 20 μm) were counted under a microscope. The colony formation assay was performed in triplicate.

### Capillary tube formation assay

BAECs were starved for 5 h before seeding 1.5×10^4^ cells onto growth factor-reduced Matrigel-coated 96-well plates (Nalge Nunc Int. Corp., Rochester, NY), and were incubated in a conditioned medium harvested from transfected Huh7 cells at 37°C in 5% CO_2_ for 6 h. The cells were photographed under a light microscope (magnification, ×100). Tube formation was quantified by counting number of branches per microscopic field in 3 randomly selected fields using Image J software (NIH).

### Xenograft mouse model

Animal studies were conducted in accordance with the institutional guidelines for care and use of laboratory animals. A subcutaneous xenograft tumor model was previously established in BALB/c nu/nu mice using shCon- or shGα_12_-SK-Hep1 cells (*n* = 8-9) [13].

### Immunohistochemistry

The tumor tissue sections were subjected to immunohistochemistry. Tumor xenografts were fixed in 10% formalin, and then embedded in paraffin. The 4-μm-thick tissue sections were immunostained with antibodies of interest.

### TUNEL assay

TUNEL assay was carried out using the DeadEnd Colorimetric TUNEL System (Promega, Madison), according to the manufacturer's instruction.

### Statistical analysis

Data were shown as the mean±S.E. from at least three independent experiments. Statistical significance was assessed using SPSS 20.0 by one-way analysis of variance procedures and Student's *t*-test. The Kaplan-Meier method was used for survival analysis. The log-rank test and/or Breslow test were used to compare survival between groups. Chi-square tests were used to compare the categorical variables. Coefficients of correlation were determined by the Pearson or Spearman analysis. *P* values of <0.05 were considered statistically significant.

## SUPPLEMENTARY FIGURE



## Abbreviations

avg: average
BAEC: bovine aortic endothelial cells
Bcl-2: B-cell lymphoma 2
DMEM: Dulbecco's Modified Eagle's Medium
EMT: epithelial-mesenchymal transition
ERK: extracellular signal-regulated kinases
FBS: fetal bovine serum
FITC: fluorescein isothiocyanate
Gα_12_QL: active mutant of Gα_12_
GPCR: G protein-coupled receptor
HCC: hepatocellular carcinoma
HNF4α: hepatocyte nuclear factor 4α
JNK1: c-Jun N-terminal kinases-1
LPA: lysophosphatidic acid
miRNA: microRNA
mTOR: mammalian target of rapamycin
NT: non-tumorous
PARP1: poly [ADP-ribose] polymerase 1
PBS: phosphate buffered saline
PI: propidium iodide
qRT-PCR: quantitative real-time polymerase chain reaction
S1P: sphingosine-1-phosphate
STAT3: signal transducer and activator of transcription 3
TUNEL: terminal deoxynucleotidyl transferase dUTP nick end labeling
UTR: untranslated region
VEGF: vascular endothelial growth factor
WT: wild-type
