# Supplementary material for: Gα12 overexpressed in hepatocellular carcinoma reduces microRNA-122 expression via HNF4α inactivation, which causes c-Met induction
Source: Oncotarget. 2015 Apr 29;6(22):19055–69. doi: 10.18632/oncotarget.3957 (PMC4662475; doi:10.18632/oncotarget.3957)
Supplement: Supplementary file 1 [file oncotarget-06-19055-s001.pdf]

$G\alpha_{12}$ overexpressed in hepatocellular carcinoma reduces microRNA-122 expression via HNF4α inactivation, which causes c-Met induction

Supplementary Material

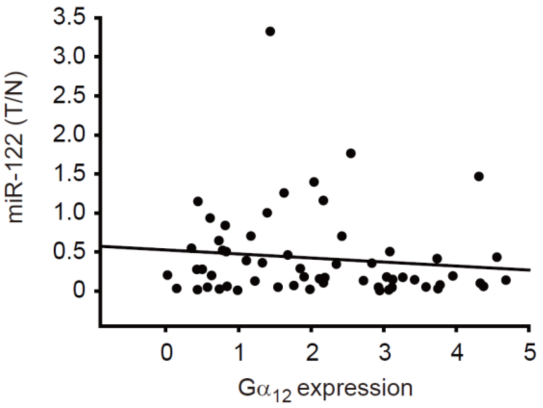

Supplementary Figure 1. Scatter plot of $G\alpha_{12}$ versus miR-122 levels in patients with HCC. Immunoblottings for $G\alpha_{12}$ and qRT-PCR assays for miR-122 were carried out on the homogenates of 59 pairs of HCC samples. There was no significant correlation between $G\alpha_{12}$ and miR-122 levels by Pearson or Spearman correlation coefficients.

Supplementary Table

Sequences of Primers

Primers	Sequences
Pri-miR-122	F: 5’-ACCCTTTCCCTTTTCAGCAT-3’<br>R: 5’-GGGAGATGAGGGGAGAGAAG-3’
Pre-miR-122	F: 5'-AGCAGAGCTGTGGAGTGTGA-3'<br>R: 5'-GCCTAGCAGTAGCTATTTAGTGTGA-3'
Mature-miR-122	5’-TGGAGTGTGACAATGGTGTTTG-3’
c-Met	F: 5’-CAGGCAGTGCAGCATGTAGT-3’<br>R: 5’-GATGATTCCCTCGGTCAGAA-3’
GNA12	F: 5’-CTCAAGGGCTCAAGGGTTCTT-3’<br>R: 5’-CAGGAACATCCCATGCTTCTC-3’
HNF4α	F: 5’-CAGGTGTTGACGATGGGCAATG-3’<br>R: 5’-AGCGGCACTGGTTCCTCTTG-3’
β-Actin	F: 5’-ACCCACACTGTGCCCATCTAC-3’<br>R: 5’-TCGGTGAGGATCTTCATGAGGTA-3’
